# Supplementary material for: Glycolytic Disruption Triggers Interorgan Signaling to Nonautonomously Restrict Drosophila Larval Growth
Source: bioRxiv. 2024 Jun 9:2024.06.06.597835. Preprint. [Version 2] doi: 10.1101/2024.06.06.597835 (PMC11185712; doi:10.1101/2024.06.06.597835)
Supplement: Supplement 1 — Supplementary Figure 1. Ldh and Gpdh1 expression patterns in the CNS, fat body, salivary gland, and intestine. Representative confocal images of second instar larval tissues expressing Ldh-GFPGenomic and immuno-stained with αGpdh1 antibody. DAPI is shown in blue, Ldh-GFP and Gpdh1 are represented in green and magenta, respectively. The rightmost panel displays the merged images of Ldh-GFP and Gpdh1 staining. (A-D) Dorsal side of CNS, (E-H) at body (I-L) salivary gland (M-P) gut. The scale bar in all the images represents 40 μM. The scale bar in (A) applies to (B-L) and the scale bar in (M) applies to (N,O,P). [file media-1.pdf]

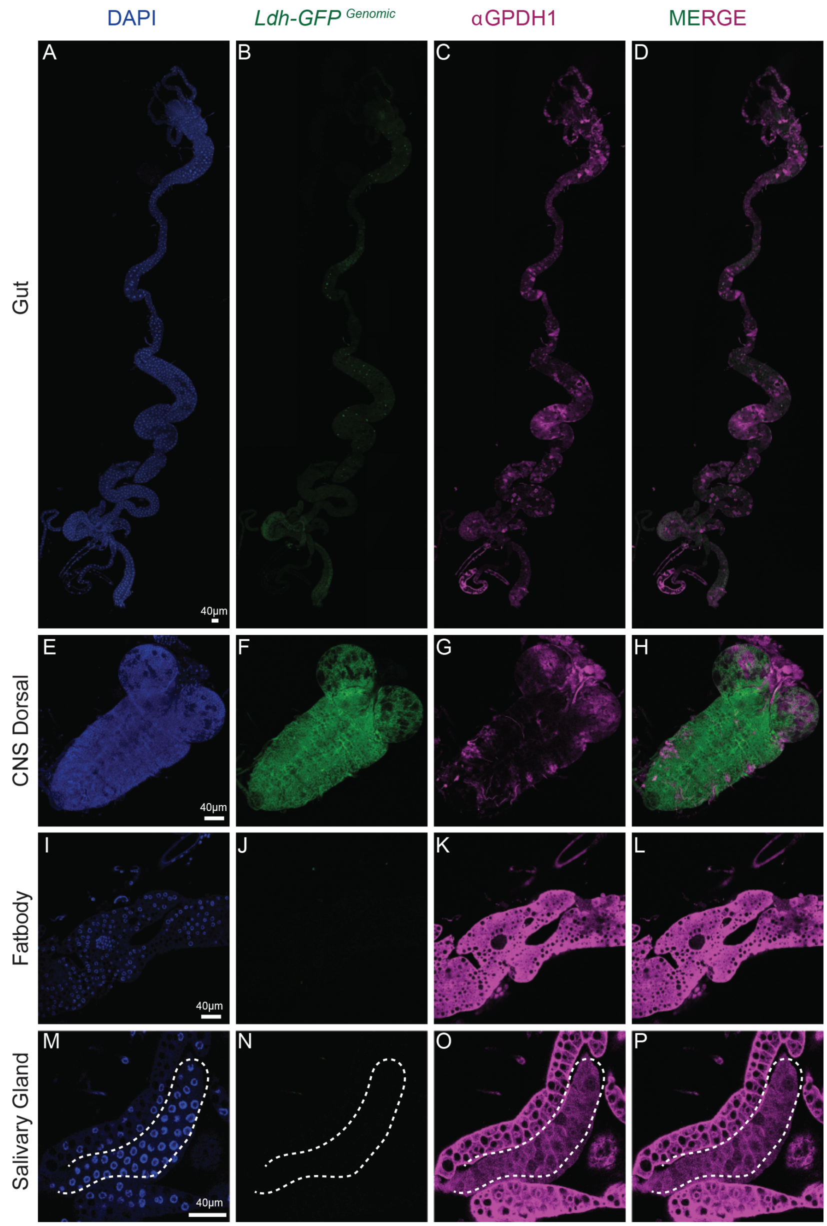

Figure S1

Supplementary Figure 1. Ldh and Gpdh1 expression patterns in the CNS, fat body, salivary gland, and intestine. Representative confocal images of second instar larval tissues expressing *Ldh-GFP*^{Genomic} and immuno-stained with αGpdh1 antibody. DAPI is shown in blue, Ldh-GFP and Gpdh1 are represented in green and magenta, respectively. The rightmost panel displays the merged images of Ldh-GFP and Gpdh1 staining. (A-D) Dorsal side of CNS, (E-H) at body (I-L) salivary gland (M-P) gut. The scale bar in all the images represents 40 μM. The scale bar in (A) applies to (B-L) and the scale bar in (M) applies to (N,O,P).
