# Supplementary material for: Glycolytic Disruption Triggers Interorgan Signaling to Nonautonomously Restrict Drosophila Larval Growth
Source: bioRxiv. 2024 Jun 9:2024.06.06.597835. Preprint. [Version 2] doi: 10.1101/2024.06.06.597835 (PMC11185712; doi:10.1101/2024.06.06.597835)
Supplement: Supplement 4 — Supplementary Figure 4. Fat body expression of a Ldh-RNAi transgene in the fat body of Gpdh1 mutants induces systemic growth defects. Growth and development of both control (r4-Gal4 and UAS-Ldh-RNAi strains) and mutant strains (Gpdh1A10/B18 mutants, r4-Ldh-RNAi, and Gpdh1A10/B18; r4-Ldh-RNAi) were monitored throughout larval development. (A) Representative images of L2 larvae (60 hr AEL) from the indicated genotypes. The scale bar represents 1 mm. (B-C) Quantification of (B) larval length and (C) time to pupation of the indicated genotypes. (B, C) All experiments are repeated a minimum of three times. n≥6 biological replicates. Error bars represent standard deviation. *P<0.05 and **P<0.01 when compared with all other genotypes. P-values were calculated using the Mann-Whitney test. [file media-4.pdf]

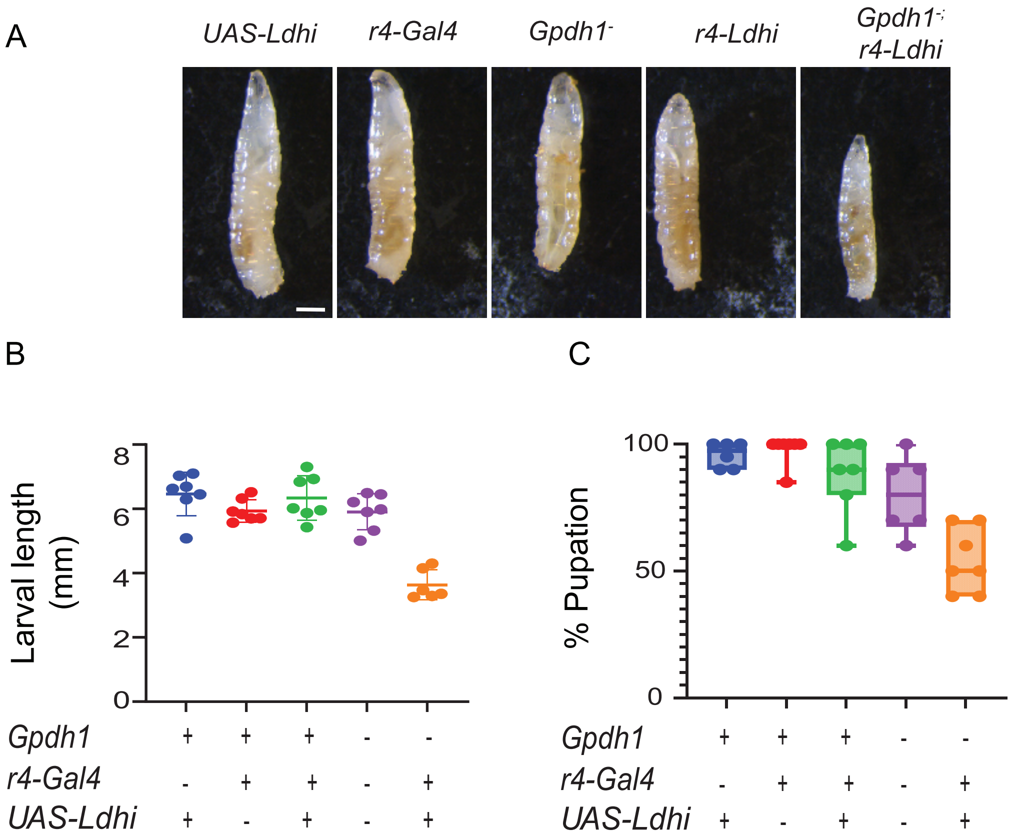

Supplementary Figure 4. Fat body expression of a *Ldh-RNAi* transgene in the fat body of *Gpdh1* mutants induces systemic growth defects. Growth and development of both control (*r4-Gal4* and *UAS-Ldh-RNAi* strains) and mutant strains (*Gpdh1*$^{A10/B18}$ mutants, *r4-Ldh-RNAi*, and *Gpdh1*$^{A10/B18}$; *r4-Ldh-RNAi*) were monitored throughout larval development. (A) Representative images of L2 larvae (60 hr AEL) from the indicated genotypes. The scale bar represents 1 mm. (B-C) Quantification of (B) larval length and (C) time to pupation of the indicated genotypes. (B, C) All experiments are repeated a minimum of three times. n≥6 biological replicates. Error bars represent standard deviation. **P*<0.05 and ***P*<0.01 when compared with all other genotypes. *P*-values were calculated using the Mann-Whitney test.
