# Supplementary material for: Glycolytic Disruption Triggers Interorgan Signaling to Nonautonomously Restrict Drosophila Larval Growth
Source: bioRxiv. 2024 Jun 9:2024.06.06.597835. Preprint. [Version 2] doi: 10.1101/2024.06.06.597835 (PMC11185712; doi:10.1101/2024.06.06.597835)
Supplement: Supplement 5 — Supplementary Figure 5. RNA-seq analysis of Ldh mutants, Gpdh1 mutants, and Gpdh1, Ldh double mutants. Volcano plot depicting the transcriptomic profiles of (A) Ldh mutants (Ldh16/17), (B) Gpdh1 mutants (Gpdh1A10/B18), and (C) double mutants (Gpdh1A10/B18; Ldh16/17) relative to the respective heterozygous control strains. n=3 biological replicates analyzed per genotype. Each sample contained 20 mid-L2 larvae. Vertical axis indicates −log10(FDR) and horizontal axis represents log(FC). The significantly upregulated genes are shown in yellow and downregulated are shown in black. FDR- fold discovery rate and FC-fold change. [file media-5.pdf]

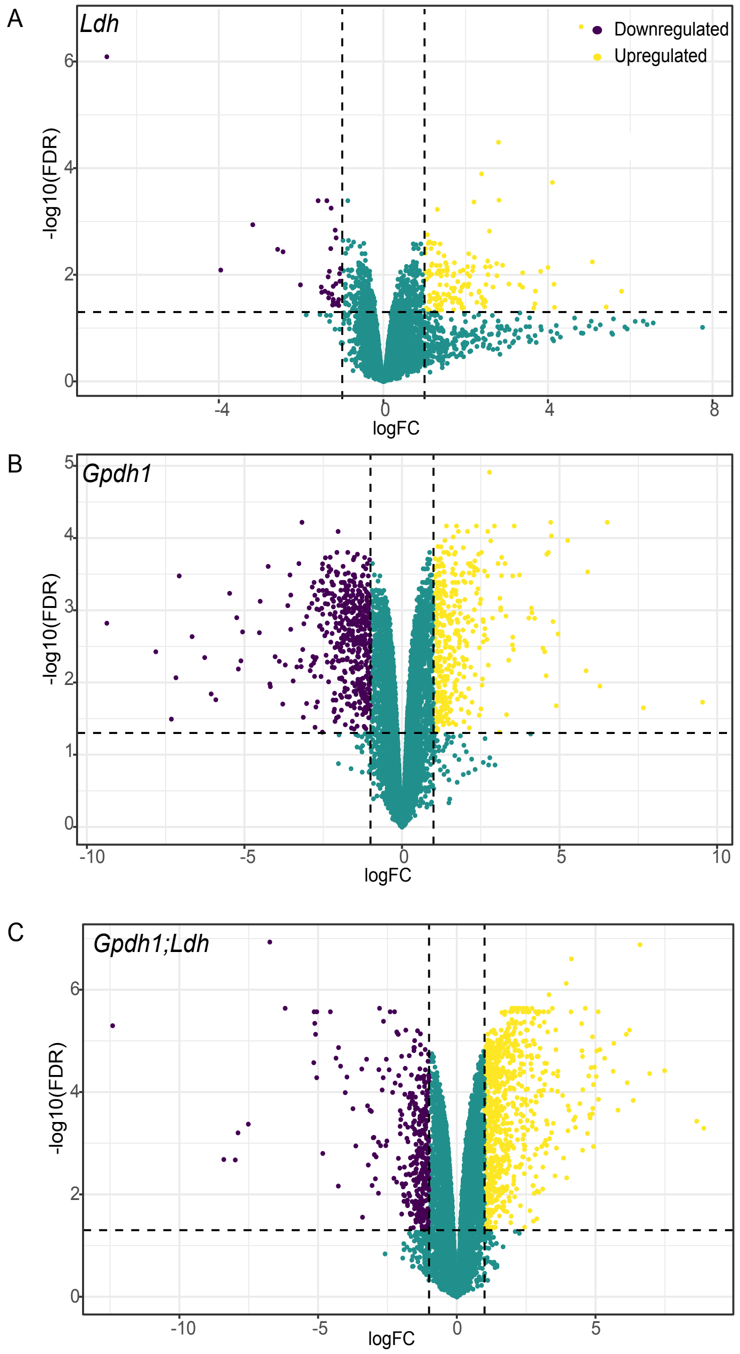

Supplementary Figure 5. RNA-seq analysis of *Ldh* mutants, *Gpdh1* mutants, and *Gpdh1, Ldh* double mutants. Volcano plot depicting the transcriptomic profiles of (A) *Ldh* mutants (*Ldh*$^{16/17}$), (B) *Gpdh1* mutants (*Gpdh1*$^{A10/B18}$), and (C) double mutants (*Gpdh1*$^{A10/B18}$; *Ldh*$^{16/17}$) relative to the respective heterozygous control strains. n=3 biological replicates analyzed per genotype. Each sample contained 20 mid-L2 larvae. Vertical axis indicates -log10(FDR) and horizontal axis represents log(FC). The significantly upregulated genes are shown in yellow and downregulated are shown in black. FDR- fold discovery rate and FC-fold change.
