# Supplementary material for: Glycolytic Disruption Triggers Interorgan Signaling to Nonautonomously Restrict Drosophila Larval Growth
Source: bioRxiv. 2024 Jun 9:2024.06.06.597835. Preprint. [Version 2] doi: 10.1101/2024.06.06.597835 (PMC11185712; doi:10.1101/2024.06.06.597835)
Supplement: Supplement 7 — Supplementary Figure 7. Stat-GFP expression is increased in larval tissues of Gpdh1; Ldh double mutants. (A-L) Representative confocal images of (A-D) fat body, (E-H) salivary glands and (I-L) muscles showing Stat-GFP expression in control, Gpdh1A10/B18, Ldh16/17 and Gpdh1A10/B18; Ldh16/17 double mutants. The scale bar represents 40 μM. The scale bar in (A) applies to (B-L). [file media-7.pdf]

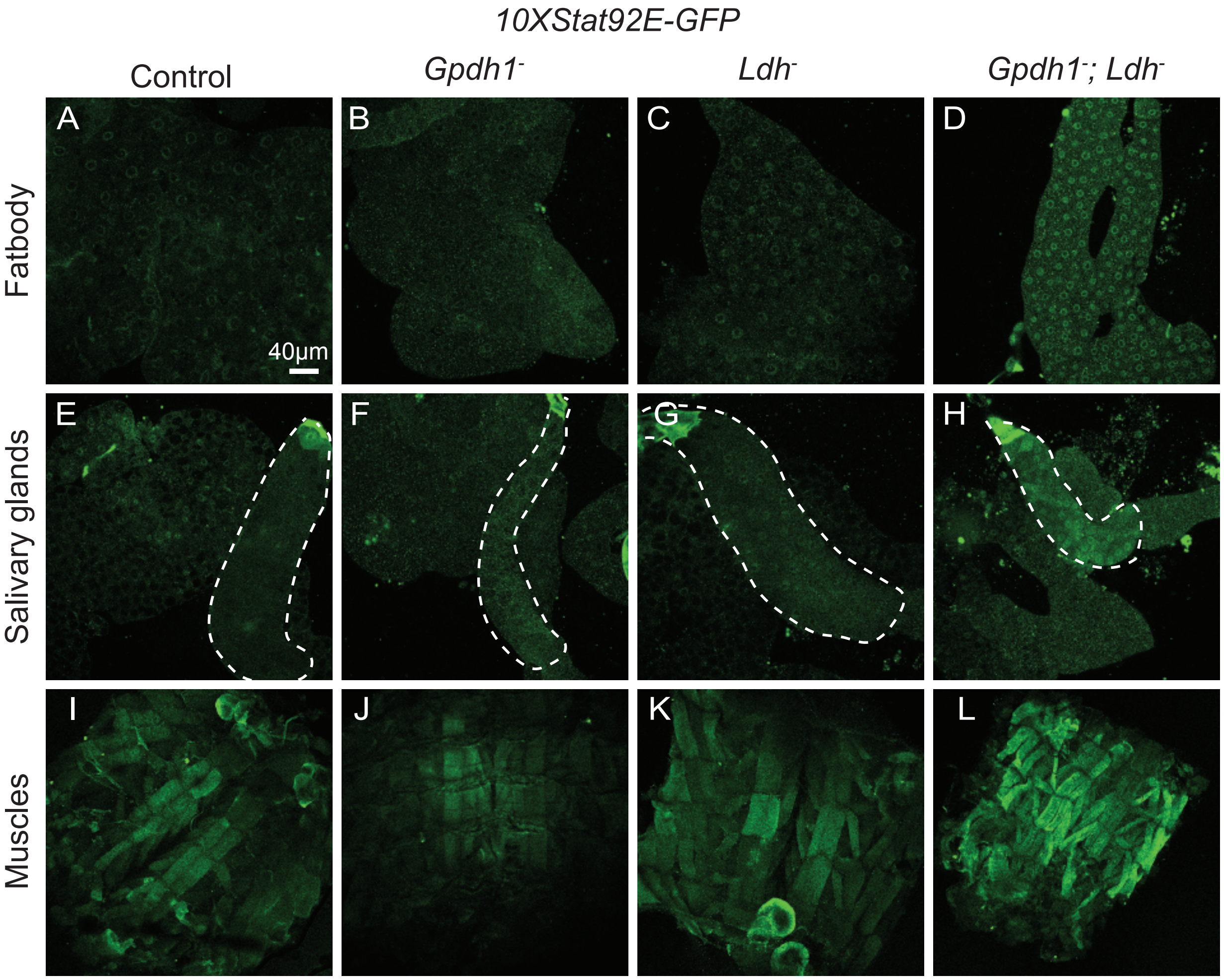

Supplementary Figure 7. *Stat-GFP* expression is increased in larval tissues of *Gpdh1; Ldh* double mutants. (A-L) Representative confocal images of (A-D) fat body, (E-H) salivary glands and (I-L) muscles showing *Stat-GFP* expression in control, *Gpdh1*$^{A10/B18}$, *Ldh*$^{16/17}$ and *Gpdh1*$^{A10/B18}$; *Ldh*$^{16/17}$ double mutants. The scale bar represents 40 µM. The scale bar in (A) applies to (B-L).
